# Tetra­kis(1-benzyl-1*H*-imidazole)dichlorido­nickel(II)

**DOI:** 10.1107/S1600536809014494

**Published:** 2009-04-25

**Authors:** Hongjuan Li, Jufeng Sun, Xianping Dai

**Affiliations:** a School of Pharmacy, Binzhou Medical College, Yantai 264003, People’s Republic of China

## Abstract

In the title compound, [NiCl_2_(C_10_H_10_N_2_)_4_], the Ni^II^ ion is located on an inversion center being coordinated by four N atoms from two pairs of symmetry-related 1-benzyl-1*H*-imidazole ligands and two chloride anions in a distorted octa­hedral geometry. Weak inter­molecular C—H⋯Cl hydrogen bonds link the mol­ecules into layers parallel to the *ab* plane.

## Related literature

For general background to crystal engineering, see: Balamurugan *et al.* (2004 ▶); Desiraju (2007 ▶); Moulton & Zaworotko (2001 ▶). For applications of imidazole derivatives, see Lu *et al.* (2006 ▶); Huang *et al.* (2006 ▶). For details of the synthesis, see Owen *et al.* (2006 ▶).
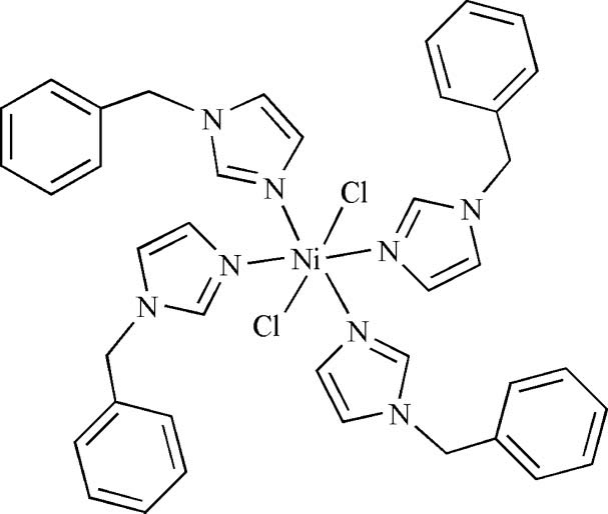


## Experimental

### 

#### Crystal data

[NiCl_2_(C_10_H_10_N_2_)_4_]
*M* *_r_* = 762.41
Orthorhombic, 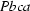

*a* = 7.296 (3) Å
*b* = 17.117 (4) Å
*c* = 29.651 (3) Å
*V* = 3703 (2) Å^3^
*Z* = 4
Mo *K*α radiation
μ = 0.71 mm^−1^
*T* = 292 K
0.48 × 0.32 × 0.30 mm

#### Data collection

Enraf–Nonius CAD-4 diffractometer
Absorption correction: spherical (modified interpolation procedure; Dwiggins, 1975 ▶) *T*_min_ = 0.743, *T*_max_ = 0.745
4699 measured reflections
3207 independent reflections
1518 reflections with *I* > 2σ(*I*)
*R*_int_ = 0.011
3 standard reflections every 200 reflections intensity decay: 1.8%

#### Refinement

*R*[*F* ^2^ > 2σ(*F* ^2^)] = 0.079
*wR*(*F* ^2^) = 0.231
*S* = 1.14
3207 reflections
220 parameters
1 restraint
H-atom parameters constrained
Δρ_max_ = 0.72 e Å^−3^
Δρ_min_ = −1.42 e Å^−3^

### 

Data collection: *DIFRAC* (Gabe & White, 1993 ▶); cell refinement: *DIFRAC*; data reduction: *NRCVAX* (Gabe *et al.*, 1989 ▶); program(s) used to solve structure: *SHELXS97* (Sheldrick, 2008 ▶); program(s) used to refine structure: *SHELXL97* (Sheldrick, 2008 ▶); molecular graphics: *ORTEPIII* (Burnett & Johnson, 1996 ▶); software used to prepare material for publication: *SHELXL97* and *PLATON* (Spek, 2009 ▶).

## Supplementary Material

Crystal structure: contains datablocks global, I. DOI: 10.1107/S1600536809014494/cv2549sup1.cif

Structure factors: contains datablocks I. DOI: 10.1107/S1600536809014494/cv2549Isup2.hkl

Additional supplementary materials:  crystallographic information; 3D view; checkCIF report

## Figures and Tables

**Table 1:** Hydrogen-bond geometry (Å, °)

*D*—H⋯*A*	*D*—H	H⋯*A*	*D*⋯*A*	*D*—H⋯*A*
C1—H1⋯Cl1^i^	0.93	2.77	3.617 (8)	151
C14—H14*B*⋯Cl1^i^	0.97	2.68	3.579 (8)	153
C13—H13⋯Cl1^ii^	0.93	2.71	3.561 (9)	152

Symmetry codes: (i) 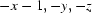; (ii) 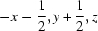.

**Fig. 1.**
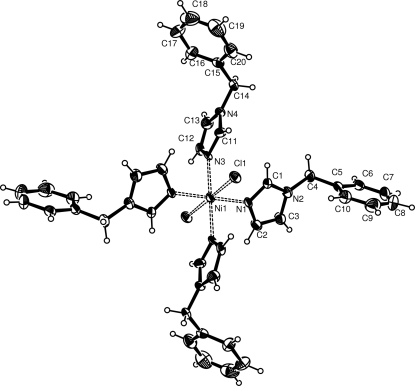
The molecular structure of (I), showing 30% probability displacement ellipsoids and the atomic numbering. The unlabelled atoms are related with the labelled ones by symmetry element (-x, -y, -z).

## supplementary crystallographic information

### Comment

Crystal engineering, the rational design of functional molecular solids, is currently an active area of investigation because of its importance in supramolecular chemistry, materials science, and solid-state chemistry (Desiraju, 2007; Moulton & Zaworotko, 2001). It is noteworthy that a promising strategy for inorganic crystal engineering through combining non-covalent bonds such as van der Waals, π···π stacking and hydrogen bonds with coordination chemistry has attracted increasing attention in recent years (Balamurugan *et al.*, 2004). Organic ligands are often used to coordinate to transition metals *via* coordinate bonds to generate metal complexes as the building blocks of the assembly. Imidazole and its derivatives are ubiquitous in biological and biochemical structure and function and thus attracted special attention in the construction of some interesting metal-organic frameworks in recent years (Huang *et al.*, 2006; Lu *et al.*, 2006). Here, we report the crystal structure of the title compound, (I).

In (I) (Fig. 1), each Ni^II^ ion displays a slightly distorted octahedral coordination geometry defined by four 1-benzyl-1*H*-imidazole ligands and two chloride anions. The Ni—N bond lengths are in the range of 2.070 (5) Å to 2.140 (3) Å and the Ni—Cl bond lengths is 2.468 (2) Å. Weak intermolecular C—H···Cl hydrogen bonds (Table 1) enhance the crystal packing stability.

### Experimental

The 1-benzyl-1*H*-imidazole was prepared according to the literature (Owen *et al.*, 2006). Nickel (II) chloride hexahydrate (1 mmol, 0.24 g) and 1-benzyl-1*H*-imidazole (4 mmol, 0.63 g) were mixed in chloroform (15 ml) and the mixture was stirred for 5 h at room temperature. After filtration, the solid was dissolved in methanol (8 ml). Green crystals suitable for X-ray analysis were obtained by slow evaporation of this solution over a period of six days.

### Refinement

All H atoms were positioned geometrically with C—H = 0.93 and 0.97 Å, and refined in the riding model approximation, with U_iso_(H) = 1.2 U_eq_(C) .

### Crystal data

**Table:**

[NiCl_2_(C_10_H_10_N_2_)_4_]	*F*(000) = 1592
*M**_r_* = 762.41	*D*_x_ = 1.368 Mg m^−^^3^
Orthorhombic, *P**b**c**a*	Mo *K*α radiation, λ = 0.71073 Å
Hall symbol: -P 2ac 2ab	Cell parameters from 20 reflections
*a* = 7.296 (3) Å	θ = 5.7–6.9°
*b* = 17.117 (4) Å	µ = 0.71 mm^−^^1^
*c* = 29.651 (3) Å	*T* = 292 K
*V* = 3703 (2) Å^3^	Block, green
*Z* = 4	0.48 × 0.32 × 0.30 mm

### Data collection

**Table:**

Enraf–Nonius CAD-4 diffractometer	1518 reflections with *I* > 2σ(*I*)
Radiation source: fine-focus sealed tube	*R*_int_ = 0.011
graphite	θ_max_ = 25.6°, θ_min_ = 2.8°
ω/2θ scans	*h* = −1→8
Absorption correction: for a sphere (modified interpolation procedure; Dwiggins, 1975)	*k* = −2→20
*T*_min_ = 0.743, *T*_max_ = 0.745	*l* = −9→35
4699 measured reflections	3 standard reflections every 200 reflections
3207 independent reflections	intensity decay: 1.9%

### Refinement

**Table:**

Refinement on *F*^2^	Primary atom site location: structure-invariant direct methods
Least-squares matrix: full	Secondary atom site location: difference Fourier map
*R*[*F*^2^ > 2σ(*F*^2^)] = 0.079	Hydrogen site location: inferred from neighbouring sites
*wR*(*F*^2^) = 0.231	H-atom parameters constrained
*S* = 1.14	*w* = 1/[σ^2^(*F*_o_^2^) + (0.0948*P*)^2^] where *P* = (*F*_o_^2^ + 2*F*_c_^2^)/3
3207 reflections	(Δ/σ)_max_ < 0.001
220 parameters	Δρ_max_ = 0.72 e Å^−^^3^
1 restraint	Δρ_min_ = −1.42 e Å^−^^3^

### Special details

**Table:**

Experimental. In spherical absoprtion correction, interpolation using Int.Tab. Vol. C (1992) p. 523,Tab. 6.3.3.3 for values of muR in the range 0–2.5, and Int.Tab. Vol.II (1959) p.302; Table 5.3.6 B for muR in the range 2.6–10.0, was used. The interpolation procedure of C.W.Dwiggins Jr (Acta Cryst.(1975) A31,146–148) was used with some modification.The most probable reason for a large number of missing reflections - 238 - lies in the fact that it is very difficult to obtain high quality crystal. After we collected the reflections of the crystal, we couldn't gain perfect crystal data. To get better and reasonable structure of the crystal, those reflections which seriously influence the optimization of the crystal structure were omitted during the course of refinement of the data.
Geometry. All e.s.d.'s (except the e.s.d. in the dihedral angle between two l.s. planes) are estimated using the full covariance matrix. The cell e.s.d.'s are taken into account individually in the estimation of e.s.d.'s in distances, angles and torsion angles; correlations between e.s.d.'s in cell parameters are only used when they are defined by crystal symmetry. An approximate (isotropic) treatment of cell e.s.d.'s is used for estimating e.s.d.'s involving l.s. planes.
Refinement. Refinement of *F*^2^ against ALL reflections. The weighted *R*-factor *wR* and goodness of fit *S* are based on *F*^2^, conventional *R*-factors *R* are based on *F*, with *F* set to zero for negative *F*^2^. The threshold expression of *F*^2^ > σ(*F*^2^) is used only for calculating *R*-factors(gt) *etc*. and is not relevant to the choice of reflections for refinement. *R*-factors based on *F*^2^ are statistically about twice as large as those based on *F*, and *R*- factors based on ALL data will be even larger.

### Fractional atomic coordinates and isotropic or equivalent isotropic displacement parameters (Å^2^)

**Table:**

	*x*	*y*	*z*	*U*_iso_*/*U*_eq_	
Ni1	0.0000	0.0000	0.0000	0.0340 (4)	
Cl1	−0.2771 (3)	−0.07775 (10)	−0.01632 (6)	0.0466 (5)	
N1	−0.1100 (8)	0.0385 (3)	0.06038 (16)	0.0359 (13)	
N2	−0.2920 (9)	0.0980 (3)	0.10849 (18)	0.0471 (16)	
N3	−0.1313 (8)	0.0963 (3)	−0.03282 (17)	0.0408 (14)	
N4	−0.3505 (9)	0.1751 (3)	−0.05553 (17)	0.0458 (16)	
C1	−0.2707 (11)	0.0710 (4)	0.0662 (2)	0.0459 (18)	
H1	−0.3595	0.0749	0.0438	0.055*	
C2	−0.0234 (12)	0.0452 (5)	0.1010 (2)	0.053 (2)	
H2	0.0941	0.0271	0.1073	0.063*	
C3	−0.1335 (12)	0.0818 (5)	0.1306 (2)	0.057 (2)	
H3	−0.1062	0.0938	0.1604	0.068*	
C4	−0.4477 (13)	0.1410 (5)	0.1252 (2)	0.062 (3)	
H4A	−0.5317	0.1503	0.1004	0.075*	
H4B	−0.4061	0.1914	0.1360	0.075*	
C5	−0.5503 (11)	0.1003 (5)	0.1627 (2)	0.048 (2)	
C6	−0.6202 (12)	0.1452 (5)	0.1977 (2)	0.058 (2)	
H6	−0.5998	0.1988	0.1988	0.069*	
C7	−0.7196 (15)	0.1086 (8)	0.2304 (3)	0.090 (4)	
H7	−0.7623	0.1384	0.2545	0.107*	
C8	−0.7590 (18)	0.0333 (9)	0.2301 (3)	0.102 (4)	
H8	−0.8334	0.0117	0.2523	0.123*	
C9	−0.6870 (18)	−0.0131 (6)	0.1959 (4)	0.088 (3)	
H9	−0.7085	−0.0666	0.1956	0.106*	
C10	−0.5826 (14)	0.0214 (5)	0.1620 (3)	0.068 (3)	
H10	−0.5346	−0.0091	0.1389	0.081*	
C11	−0.3010 (12)	0.1020 (4)	−0.0462 (2)	0.0489 (19)	
H11	−0.3794	0.0594	−0.0489	0.059*	
C12	−0.0682 (11)	0.1720 (4)	−0.0329 (2)	0.0479 (19)	
H12	0.0495	0.1871	−0.0246	0.057*	
C13	−0.2007 (13)	0.2212 (5)	−0.0467 (2)	0.057 (2)	
H13	−0.1926	0.2752	−0.0496	0.068*	
C14	−0.5292 (11)	0.2037 (5)	−0.0674 (2)	0.059 (2)	
H14A	−0.5335	0.2594	−0.0614	0.071*	
H14B	−0.6191	0.1789	−0.0480	0.071*	
C15	−0.5830 (8)	0.1897 (3)	−0.11593 (13)	0.051 (2)	
C16	−0.4895 (7)	0.2276 (3)	−0.15044 (17)	0.069 (2)	
H16	−0.3905	0.2597	−0.1437	0.083*	
C17	−0.5439 (10)	0.2173 (4)	−0.19497 (14)	0.091 (4)	
H17	−0.4814	0.2426	−0.2181	0.109*	
C18	−0.6919 (10)	0.1692 (4)	−0.20499 (17)	0.106 (4)	
H18	−0.7283	0.1623	−0.2348	0.127*	
C19	−0.7854 (8)	0.1313 (4)	−0.1705 (3)	0.102 (4)	
H19	−0.8844	0.0991	−0.1772	0.122*	
C20	−0.7310 (8)	0.1416 (3)	−0.1260 (2)	0.071 (3)	
H20	−0.7935	0.1163	−0.1029	0.086*	

### Atomic displacement parameters (Å^2^)

**Table:**

	*U*^11^	*U*^22^	*U*^33^	*U*^12^	*U*^13^	*U*^23^
Ni1	0.0249 (7)	0.0408 (7)	0.0363 (6)	0.0012 (6)	0.0032 (6)	−0.0016 (5)
Cl1	0.0319 (11)	0.0484 (11)	0.0597 (9)	−0.0045 (8)	−0.0011 (8)	−0.0002 (8)
N1	0.019 (3)	0.050 (3)	0.039 (3)	0.001 (3)	0.006 (3)	−0.002 (2)
N2	0.044 (4)	0.051 (4)	0.046 (3)	0.013 (3)	0.008 (3)	−0.004 (3)
N3	0.013 (3)	0.069 (4)	0.040 (3)	0.007 (3)	0.000 (3)	0.006 (3)
N4	0.042 (4)	0.053 (4)	0.042 (3)	0.026 (3)	0.006 (3)	0.005 (3)
C1	0.037 (5)	0.068 (5)	0.033 (3)	0.007 (4)	0.007 (3)	0.005 (3)
C2	0.037 (5)	0.072 (5)	0.049 (4)	0.014 (4)	−0.001 (4)	−0.008 (4)
C3	0.043 (5)	0.081 (6)	0.047 (4)	−0.001 (4)	−0.002 (4)	−0.011 (4)
C4	0.059 (7)	0.068 (6)	0.060 (5)	0.028 (4)	0.024 (4)	0.009 (4)
C5	0.033 (5)	0.071 (6)	0.040 (4)	0.014 (4)	0.001 (3)	−0.005 (3)
C6	0.045 (6)	0.084 (6)	0.045 (4)	0.019 (5)	−0.004 (4)	−0.004 (4)
C7	0.057 (7)	0.161 (11)	0.051 (5)	0.022 (7)	0.019 (5)	0.012 (6)
C8	0.076 (10)	0.147 (11)	0.084 (7)	0.005 (8)	0.033 (7)	0.047 (8)
C9	0.083 (9)	0.077 (7)	0.104 (7)	−0.011 (6)	−0.009 (7)	0.026 (6)
C10	0.064 (7)	0.080 (6)	0.060 (5)	0.005 (5)	0.003 (5)	0.000 (4)
C11	0.050 (5)	0.059 (5)	0.037 (3)	0.012 (4)	−0.007 (4)	0.003 (3)
C12	0.031 (5)	0.059 (5)	0.054 (4)	−0.009 (4)	0.014 (4)	0.005 (4)
C13	0.057 (6)	0.056 (5)	0.057 (4)	−0.006 (4)	−0.001 (4)	0.003 (4)
C14	0.041 (6)	0.085 (6)	0.052 (4)	0.029 (4)	0.017 (4)	0.011 (4)
C15	0.042 (5)	0.052 (5)	0.058 (4)	0.013 (4)	−0.002 (4)	0.010 (3)
C16	0.072 (7)	0.078 (6)	0.057 (5)	−0.003 (5)	0.004 (5)	0.014 (4)
C17	0.100 (10)	0.128 (9)	0.046 (5)	0.016 (7)	−0.010 (6)	0.014 (5)
C18	0.119 (13)	0.114 (10)	0.086 (7)	0.041 (8)	−0.031 (8)	−0.027 (6)
C19	0.073 (9)	0.105 (9)	0.127 (9)	−0.011 (6)	−0.012 (8)	−0.035 (7)
C20	0.056 (7)	0.059 (6)	0.100 (7)	−0.003 (5)	0.009 (6)	0.001 (5)

### Geometric parameters (Å, °)

**Table:**

Ni1—N1^i^	2.070 (5)	C6—H6	0.9300
Ni1—N1	2.070 (5)	C7—C8	1.320 (15)
Ni1—N3	2.140 (6)	C7—H7	0.9300
Ni1—N3^i^	2.140 (6)	C8—C9	1.391 (15)
Ni1—Cl1	2.468 (2)	C8—H8	0.9300
Ni1—Cl1^i^	2.468 (2)	C9—C10	1.392 (13)
N1—C1	1.310 (9)	C9—H9	0.9300
N1—C2	1.365 (8)	C10—H10	0.9300
N2—C1	1.345 (8)	C11—H11	0.9300
N2—C3	1.358 (10)	C12—C13	1.345 (11)
N2—C4	1.441 (9)	C12—H12	0.9300
N3—C11	1.304 (9)	C13—H13	0.9300
N3—C12	1.376 (8)	C14—C15	1.512 (8)
N4—C11	1.333 (8)	C14—H14A	0.9700
N4—C13	1.373 (10)	C14—H14B	0.9700
N4—C14	1.436 (9)	C15—C16	1.3900
C1—H1	0.9300	C15—C20	1.3900
C2—C3	1.345 (10)	C16—C17	1.3900
C2—H2	0.9300	C16—H16	0.9300
C3—H3	0.9300	C17—C18	1.3900
C4—C5	1.510 (10)	C17—H17	0.9300
C4—H4A	0.9700	C18—C19	1.3900
C4—H4B	0.9700	C18—H18	0.9300
C5—C10	1.371 (10)	C19—C20	1.3900
C5—C6	1.389 (9)	C19—H19	0.9300
C6—C7	1.365 (13)	C20—H20	0.9300
			
N1^i^—Ni1—N1	180.0	C5—C6—H6	120.9
N1^i^—Ni1—N3	91.4 (2)	C8—C7—C6	124.0 (10)
N1—Ni1—N3	88.6 (2)	C8—C7—H7	118.0
N1^i^—Ni1—N3^i^	88.6 (2)	C6—C7—H7	118.0
N1—Ni1—N3^i^	91.4 (2)	C7—C8—C9	118.7 (10)
N3—Ni1—N3^i^	180.0	C7—C8—H8	120.6
N1^i^—Ni1—Cl1	88.65 (16)	C9—C8—H8	120.6
N1—Ni1—Cl1	91.35 (16)	C8—C9—C10	119.4 (10)
N3—Ni1—Cl1	87.67 (17)	C8—C9—H9	120.3
N3^i^—Ni1—Cl1	92.33 (17)	C10—C9—H9	120.3
N1^i^—Ni1—Cl1^i^	91.35 (16)	C5—C10—C9	120.0 (9)
N1—Ni1—Cl1^i^	88.65 (16)	C5—C10—H10	120.0
N3—Ni1—Cl1^i^	92.33 (17)	C9—C10—H10	120.0
N3^i^—Ni1—Cl1^i^	87.67 (17)	N3—C11—N4	113.0 (7)
Cl1—Ni1—Cl1^i^	180.0	N3—C11—H11	123.5
C1—N1—C2	105.2 (6)	N4—C11—H11	123.5
C1—N1—Ni1	126.6 (5)	C13—C12—N3	110.4 (8)
C2—N1—Ni1	127.6 (5)	C13—C12—H12	124.8
C1—N2—C3	106.3 (6)	N3—C12—H12	124.8
C1—N2—C4	125.9 (7)	C12—C13—N4	105.7 (7)
C3—N2—C4	127.6 (6)	C12—C13—H13	127.1
C11—N3—C12	104.3 (6)	N4—C13—H13	127.1
C11—N3—Ni1	128.4 (5)	N4—C14—C15	114.5 (6)
C12—N3—Ni1	125.2 (5)	N4—C14—H14A	108.6
C11—N4—C13	106.5 (7)	C15—C14—H14A	108.6
C11—N4—C14	128.1 (7)	N4—C14—H14B	108.6
C13—N4—C14	125.0 (7)	C15—C14—H14B	108.6
N1—C1—N2	111.9 (6)	H14A—C14—H14B	107.6
N1—C1—H1	124.1	C16—C15—C20	120.0
N2—C1—H1	124.1	C16—C15—C14	120.0 (5)
C3—C2—N1	109.8 (7)	C20—C15—C14	119.9 (5)
C3—C2—H2	125.1	C17—C16—C15	120.0
N1—C2—H2	125.1	C17—C16—H16	120.0
C2—C3—N2	106.8 (6)	C15—C16—H16	120.0
C2—C3—H3	126.6	C18—C17—C16	120.0
N2—C3—H3	126.6	C18—C17—H17	120.0
N2—C4—C5	114.1 (6)	C16—C17—H17	120.0
N2—C4—H4A	108.7	C17—C18—C19	120.0
C5—C4—H4A	108.7	C17—C18—H18	120.0
N2—C4—H4B	108.7	C19—C18—H18	120.0
C5—C4—H4B	108.7	C20—C19—C18	120.0
H4A—C4—H4B	107.6	C20—C19—H19	120.0
C10—C5—C6	119.6 (8)	C18—C19—H19	120.0
C10—C5—C4	121.8 (7)	C19—C20—C15	120.0
C6—C5—C4	118.5 (8)	C19—C20—H20	120.0
C7—C6—C5	118.2 (9)	C15—C20—H20	120.0
C7—C6—H6	120.9		
			
N1^i^—Ni1—N1—C1	−158 (20)	N2—C4—C5—C10	−40.6 (12)
N3—Ni1—N1—C1	35.5 (6)	N2—C4—C5—C6	142.2 (8)
N3^i^—Ni1—N1—C1	−144.5 (6)	C10—C5—C6—C7	−0.1 (12)
Cl1—Ni1—N1—C1	−52.1 (6)	C4—C5—C6—C7	177.2 (8)
Cl1^i^—Ni1—N1—C1	127.9 (6)	C5—C6—C7—C8	−2.4 (15)
N1^i^—Ni1—N1—C2	32 (22)	C6—C7—C8—C9	3.8 (18)
N3—Ni1—N1—C2	−135.0 (6)	C7—C8—C9—C10	−2.7 (18)
N3^i^—Ni1—N1—C2	45.0 (6)	C6—C5—C10—C9	0.9 (13)
Cl1—Ni1—N1—C2	137.4 (6)	C4—C5—C10—C9	−176.2 (9)
Cl1^i^—Ni1—N1—C2	−42.6 (6)	C8—C9—C10—C5	0.4 (16)
N1^i^—Ni1—N3—C11	98.3 (6)	C12—N3—C11—N4	1.1 (7)
N1—Ni1—N3—C11	−81.7 (6)	Ni1—N3—C11—N4	165.0 (4)
N3^i^—Ni1—N3—C11	−77 (56)	C13—N4—C11—N3	−1.2 (8)
Cl1—Ni1—N3—C11	9.8 (6)	C14—N4—C11—N3	−174.3 (6)
Cl1^i^—Ni1—N3—C11	−170.2 (6)	C11—N3—C12—C13	−0.6 (8)
N1^i^—Ni1—N3—C12	−100.9 (5)	Ni1—N3—C12—C13	−165.1 (5)
N1—Ni1—N3—C12	79.1 (5)	N3—C12—C13—N4	−0.1 (8)
N3^i^—Ni1—N3—C12	84 (56)	C11—N4—C13—C12	0.8 (8)
Cl1—Ni1—N3—C12	170.5 (5)	C14—N4—C13—C12	174.2 (6)
Cl1^i^—Ni1—N3—C12	−9.5 (5)	C11—N4—C14—C15	−79.1 (9)
C2—N1—C1—N2	−0.2 (8)	C13—N4—C14—C15	109.0 (8)
Ni1—N1—C1—N2	−172.4 (4)	N4—C14—C15—C16	−66.5 (8)
C3—N2—C1—N1	0.4 (8)	N4—C14—C15—C20	116.2 (7)
C4—N2—C1—N1	176.0 (7)	C20—C15—C16—C17	0.0
C1—N1—C2—C3	−0.2 (9)	C14—C15—C16—C17	−177.2 (5)
Ni1—N1—C2—C3	171.9 (5)	C15—C16—C17—C18	0.0
N1—C2—C3—N2	0.4 (10)	C16—C17—C18—C19	0.0
C1—N2—C3—C2	−0.5 (9)	C17—C18—C19—C20	0.0
C4—N2—C3—C2	−176.0 (7)	C18—C19—C20—C15	0.0
C1—N2—C4—C5	117.9 (8)	C16—C15—C20—C19	0.0
C3—N2—C4—C5	−67.5 (12)	C14—C15—C20—C19	177.2 (5)

Symmetry codes: (i) −*x*, −*y*, −*z*.

### Hydrogen-bond geometry (Å, °)

**Table:**

*D*—H···*A*	*D*—H	H···*A*	*D*···*A*	*D*—H···*A*
C1—H1···Cl1^ii^	0.93	2.77	3.617 (8)	151
C14—H14B···Cl1^ii^	0.97	2.68	3.579 (8)	153
C13—H13···Cl1^iii^	0.93	2.71	3.561 (9)	152

Symmetry codes: (ii) −*x*−1, −*y*, −*z*; (iii) −*x*−1/2, *y*+1/2, *z*.
